# Effect of glucagon-like peptide-1 receptor agonists in osteoarthritis: A systematic review of pre-clinical and human studies

**DOI:** 10.1016/j.ocarto.2025.100567

**Published:** 2025-01-28

**Authors:** Jacinta Cheng, Tia Solomon, Mahnuma Estee, Flavia M. Cicuttini, Yuan Z. Lim

**Affiliations:** a Alfred Hospital, Melbourne, VIC 3004, Australia; b School of Public Health and Preventive Medicine, Monash University, Melbourne, VIC 3004, Australia

**Keywords:** GLP-1 agonists, Arthritis, Pre-clinical, Human, Systematic review

## Abstract

**Objective:**

There is significant interest in the potential of glucagon-like peptide 1 receptor agonists (GLP-1A) to improve outcomes in osteoarthritis. We systematically reviewed the evidence from pre-clinical and human studies for effect of glucagon-like peptide 1 receptor agonists (GLP-1A) in osteoarthritis.

**Method:**

Ovid Medline, Embase and CINAHL were searched (inception to November 2024) using MeSH terms and key words to identify studies examining the association between GLP-1A use and outcomes related to osteoarthritis. Risk of bias assessment and data extraction were conducted by three reviewers independently. Qualitative evidence synthesis was performed and prospectively registered on PROSPERO (CRD42024522782 and CRD42024522787).

**Results:**

This systematic review included 11 (7 pre-clinical; 4 human studies) studies. In pre-clinical studies, GLP-1A was assessed for its effect on structural (n ​= ​6); immunomodulation (n ​= ​7); analgesia (n ​= ​1) and molecular pathways in osteoarthritis (n ​= ​5). For human studies, GLP-1A were assessed for structural (n ​= ​1) and symptomatic (n ​= ​4) effects in osteoarthritis. Pre-clinical studies consistently demonstrated favourable chondroprotective and immunomodulatory effects of GLP-1A in osteoarthritis, with a dose-dependent effect, primarily driven by inhibition of NF-κB pathway. Limited human studies supported these findings in osteoarthritis.

**Conclusion:**

There are consistent signals across limited pre-clinical and human studies to support a potential favourable structural protective, immunomodulatory and analgesic effects of GLP-1A in osteoarthritis. With the growing burden of obesity, high-quality trials are needed to determine the role of GLP-1A in osteoarthritis.

## 1 Introduction

Osteoarthritis is the most common cause of disability worldwide [1]. Obesity is an important modifiable risk factors for knee osteoarthritis [2,3]. The spectrum of osteoarthritis extends as a continuum of disease from healthy joint to early osteoarthritis and then end-stage disease [2], which is treated with knee replacement to improve pain and function. Obesity related structural changes have been detected even prior to clinical symptoms [2,3]. As such, targeting obesity is important in the management of knee osteoarthritis to improve outcomes. Most guidelines recommend weight loss in those with knee and hip osteoarthritis who are overweight and obese [4,5]. Loss of ≥10 ​% of total body weight is needed for any significant effect on knee pain [6,7], but is difficult to achieve.

There is a lot of excitement about recent advances in weight loss drug therapies with the glucagon-like peptide-1 (GLP-1) agonists. GLP-1 agonists, an incretin hormone, are used in management of diabetes, but also well known for its substantial weight loss effect, with approximately 15 ​% body weight loss within 12–24 months [8,9]. Liraglutide has been shown to result in 6.4 ​% weight loss in those with obesity or overweight with a co-morbidity [10] and semaglutide with 10.9 ​% weight loss at 6 months [10]. Beyond its broad metabolic effects, GLP-1 agonists have also been shown to have cardio- and neuro-protective effects [8,9].

There is emerging evidence that GLP-1 agonist may be effective in improving outcomes in osteoarthritis. Osteoarthritis is no longer thought to be a “wear-and-tear” disease [2,3,11]. Pre-clinical studies have demonstrated inflammation-related changes in osteoarthritis cartilage, with proinflammatory cytokines seen increase in cartilage, bone, and synovium, contributing to the initiation and progression of osteoarthritis [[12], [13], [14]]. There is also evidence to suggest anti-inflammatory effects of GLP-1 agonists [9], such that mechanistically, it may be reasonable to target this inflammation pathway in osteoarthritis. Given its pleotropic effects, GLP-1 agonist has the potential to be a novel therapy for osteoarthritis. With limited data from human studies [15], we aim to systematically reviewed the combine evidence from both pre-clinical and human studies for the effect of GLP-1 agonist in osteoarthritis.

## 2 Methods

This systematic review was conducted according to the Preferred Reporting Items for Systematic Reviews and Meta-Analyses (PRISMA) guidelines [16], and prospectively registered on PROSPERO (CRD42024522782 and CRD42024522787).

### 2.1 Search strategy

Major electronic databases (Ovid Medline, Embase and CINAHL) were searched between their inception and 30^th^ November 2024 using MeSH terms and key words to identify studies examining the association between GLP-1 agonist use and any outcome measures related to arthritis. The search terms included “included “GLP-1 agonist” OR “albiglutide” OR “dulaglutide” OR “exenatide” OR “liraglutide” OR “lixisenatide” OR “semaglutide” OR “tirzepatide” AND “arthritis” OR “arthropathy” OR “joint disease”. Searches were limited to English language. All reference lists of included articles and reviews were hand searched to further identify potentially relevant studies. Pre-clinical and human studies were included: *in vitro* cell-based and *in vivo* animal studies were categorised as “pre-clinical”, and human clinical studies categorised as “human”.

### 2.2 Study selection

An initial 3-stage determination method (title then abstract screening, followed by full text screening) was used to assess the eligibility of available studies, performed by JC and FMC independently. Following full text screening, any disagreement between the 2 authors was resolved by discussion. This systematic review included any studies that directly examined the association between GLP-1 agonist use and any outcome measures related to osteoarthritis. For human studies, there were no restrictions set for the type of GLP-1 agonists, its dosage, frequency, or route of administration. For pre-clinical studies, there were no limitations set for the species or sex of animals; type, source or the phenotype of cells used, as long as it was used on an osteoarthritis disease model. Non-English articles, reviews, case reports, case studies, book chapters, conference abstracts, and studies not analysing outcomes of interest were excluded.

### 2.3 Data extraction

Data extraction for pre-clinical studies was conducted by JC and YZL independently; and for human studies by JC and ME independently, with consistency cross-checked by FMC. For pre-clinical studies, data extracted included: authors, year of publication, study design, type of arthritis model, animal characteristics (species, sex, age and weight), number of animals per group, cell source and phenotype, GLP-1 agonist therapy (name, dosage, frequency, duration, route of administration), timing of first drug administration, timing of when animals were sacrificed, duration of follow-up, outcome measures and main findings. For human studies, extracted data included: authors, year of publication, study design, study population, baseline characteristics of participants, type of arthritis, intervention and comparator, outcome measures and main findings.

### 2.4 Risk of bias assessment

Risk of bias for human studies was assessed by using Cochrane risk of bias tool (RoB2) [17] for randomised trials and risk of bias in non-randomised studies – of interventions tool (ROBINS-I) [18] for non-randomised trials and cohort studies, performed independently by MME and YZL. Risk of bias for animal studies was conducted by using the Systematic Review Centre for Laboratory Animal Experimentation (SYRCLE)'s risk of bias tool [19], executed by TS and YZL independently. Any differences in assessments were adjudicated by discussion between the two authors to reach for a consensus. The overall risk of bias for human studies was scored as low, moderate, or high.

### 2.5 Data synthesis

Results were tabulated and presented according to the type of studies: pre-clinical or human studies. Meta-analysis was precluded by the significant heterogeneity of the included studies: study population; GLP-1 agonist dose, duration and route of administration; type of arthritis; and outcome measures. As such, best-evidence qualitative synthesis was performed.

## 3 Results

### 3.1 Search results

**Fig. 1:**
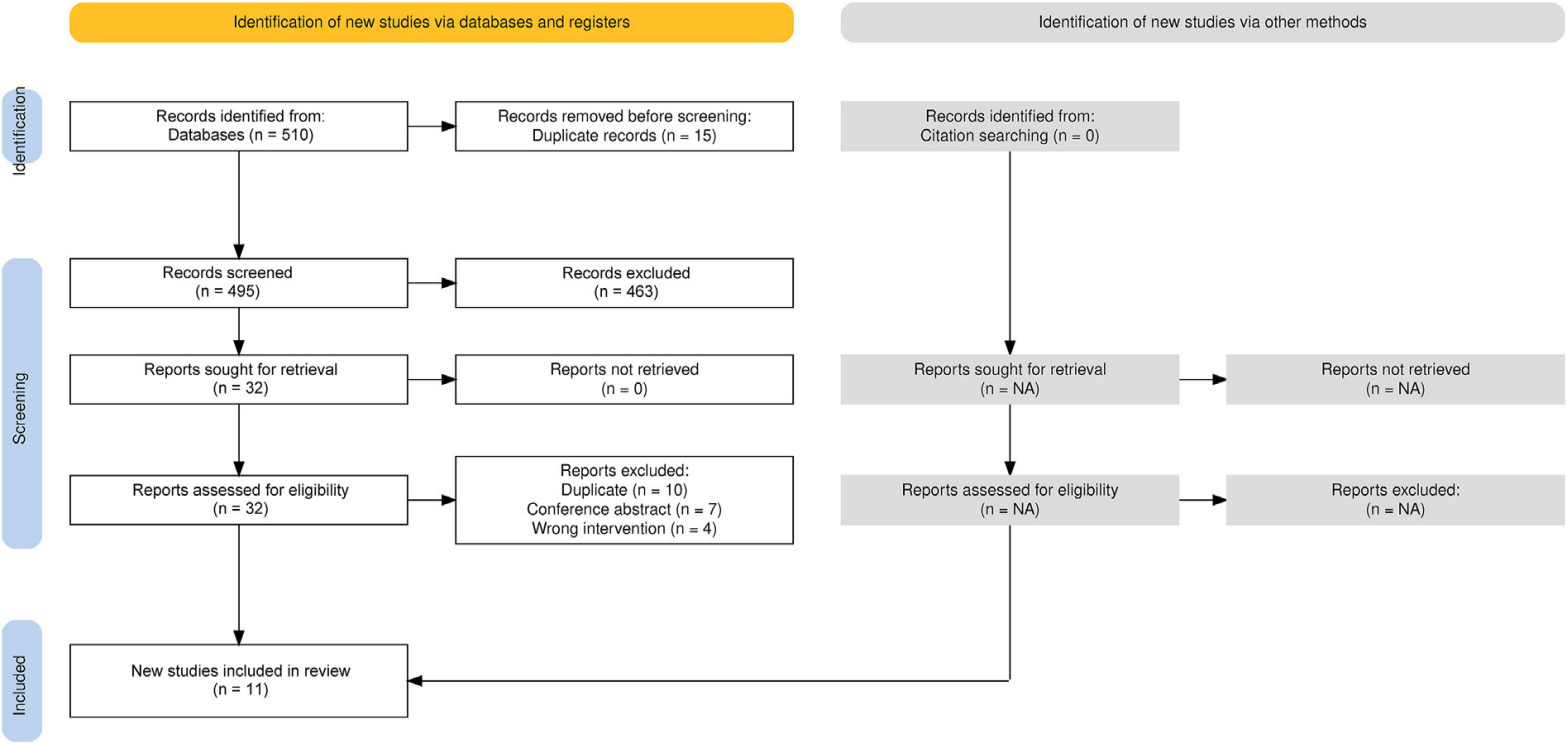
Preferred Reporting Items for Systematic Reviews and Meta-Analyses (PRISMA) flow diagram of search algorithm.

Through 3 major electronic databases, a total of 510 studies were identified. A total of 495 studies remained for title and abstract screening after removal of 15 duplicates, as shown in Fig. 1. Thirty-two studies progressed to full text screening after title and abstract screening. Twenty-one studies were excluded for reasons including: duplication, n ​= ​10; abstract only, n ​= ​7; wrong intervention, n ​= ​4). This systematic review had included a total of 11 studies (7 pre-clinical and 4 human studies).

## 4 Pre-clinical studies

### 4.1 Overall description of included studies

Seven pre-clinical studies evaluated the effect of GLP-1 agonists in osteoarthritis [[20], [21], [22], [23], [24], [25], [26]]. Two studies used both cell and animal models [20,23], one study used an animal model alone [24], and four studies used a cell model alone [21,22,25,26]. The characteristics of the seven studies categorised by the effect of GLP-1 agonists are available in Appendix 1 eTable 1.

All pre-clinical animal studies were conducted in young, healthy male mice or rats on a standard in-bred wild-type genetic background [20,23,24], by using chemically-induced [23,24] or surgically-induced [20] osteoarthritis animal models. All the cell studies used knee chondrocytes from osteoarthritis [[20], [21], [22], [23],^25^,^26^] models.

Different GLP-1 agonists were used in the pre-clinical studies. While all animal models used Liraglutide [20,23,24], a variety of GLP-1 agonists were used in cell studies: Liraglutide [20,22,23,26]; Dulaglutide [21] and Exendin-4 [25]. Significant heterogeneity was observed in dose, timing of GLP-1 agonist, duration and route of administration among the pre-clinical studies. In cell-culture studies, while Liraglutide was the most used GLP-1 agonist [20,22,23,26], its dose ranged from 53.1 ​nM to 500 ​mM. GLP-1 agonist was generally administered early (day 1) in the animal models [23,24] but one study also assessed delayed administration (day 7) in a model to evaluate the long-term effect of GLP-1 agonists [23]. The duration of GLP-1 agonist treatment ranged from 9 to 28 days in the animal studies [23,24] and 24–48 ​h in cell studies. Liraglutide was administered intra-articularly in only one animal study [23], while the remaining two animal studies [20,24] administered Liraglutide subcutaneously at 50 ​μg/kg/day.

### 4.2 Effect of GLP-1 agonists on structural outcomes

The potential effect of GLP-1 agonists on structural changes or processes related to structural pathology was evaluated in six studies [[20], [21], [22], [23],^25^,^26^] (Table 1 and Appendix 1 eTable 1). Through cell studies in human, mice or rats knee chondrocytes, GLP-1 agonists were shown to have structurally protective effects, evidenced by its ability in attenuating catabolic factors [23,26], reduction in extra-cellular matrix degrading proteins, in particular matrix metalloproteinase (MMP-3, MMP-13) [[20], [21], [22], [23],^25^,^26^] and A disintegrin and metalloproteinase with thrombospondin motifs (ADAMTS)-4 and ADAMTS-5 [22,25,26], reduction in ER-stress proteins [e.g. CCAAT-enhancer-binding-protein homologous protein (CHOP), caspase 12, Protein disulfide isomerase (PDI)] [20] while reciprocally enhancing anabolic proteins (type II collagen, aggrecan) [[20], [21], [22],^25^,^26^] in a dose-dependent effect [[20], [21], [22], [23],^25^]. Cytoprotective effects were also demonstrated by GLP-1 agonists where these chondrocytes were shown to have less apoptosis [20,26] and as such, improved viability [20,26]. Animal studies revealed Liraglutide ameliorated cartilage destruction, erosion, proteoglycan and cellular loss [20] in knee osteoarthritis rats model, such that a lower total synovitis score was observed [20,23].

**Table 1:** Effect of GLP-1 agonists on structural changes, immunomodulation and symptoms in pre-clinical studies.

Author	Cell phenotype/Animal	Mode of GLP-1 administration and type of GLP-1	Main findings	Dose dependent effect	Weight change
***Structural effect***					
**Cell studies**					
Zhang 2024 [26]	Rats knee chondrocytes (OA)		↓ catabolic factors (MMP-1, MMP-3, MMP-13, ADAMTS-4 and ADAMTS-5 ↑ Collagen II ↑ aggrecan ↓ chondrocytes apoptosis (Caspase 3)		
Meurot 2022 [23]	Mice knee chondrocyte (OA)		↓ catabolic factors and activity in chondrocytes (MMP-3, MMP-13, GAG)	Yes	
Li 2020 [21]	Human SW1353 chondrocytes (OA)		↓ PGE2 and COX-2 ↓ ECM degrading proteins (MMP-3, MMP-13) ↑ type II collagen ↑ aggrecan	Yes	
Mei 2019 [22]	Human chondrocytes (OA)		↓ ECM degrading proteins (MMP-3, MMP-13, ADAMTS-4 and ADAMTS-5) ↑ type II collagen ↑ aggrecan	Yes	
Tong 2019 [25]	Human knee chondrocytes (OA)		↓ ECM degrading proteins (MMP-3, MMP-13, ADAMTS-4 and ADAMTS-5) ↑ type II collagen ↑ aggrecan	Yes	
Chen 2018 [20]	Rats knee chondrocyte (OA)		↓ ER-stress proteins (CHOP, caspase 12, PDI, GRP78) ↓ pro-apoptotic protein (activated-caspase3, Bax) ↑ anti-apoptotic proteins (Bcl-2) ↑ chondrocytes viability ↑ ECM protein collagen II ↓ ECM degrading protein (MMP-3)	Yes	
**Animal studies**					
Meurot 2022 [23]	Mice	Intra-articular liraglutide	↓ total synovitis score		Weight loss
Chen 2018 [20]	Rats	Subcutaneous liraglutide	↓ cartilage destruction (lower OARSI scores) ↓ CHOP (ER-stress protein)		
***Immunomodulatory effect***					
**Cell studies**					
Zhang 2024 [26]	Rats knee chondrocytes (OA)		↓ proinflammatory cytokines (IL-1β, IL-6, IL-12, TNF-⍺)		
Meurot 2022 [23]	Mice knee chondrocyte (OA)		↓ proinflammatory cytokines (iNOS, COX-2, TNF-⍺) in chondrocytes	Yes	
Li 2020 [21]	Human SW1353 chondrocytes (OA)		↓ proinflammatory cytokines (IL-6, IL-8 and MCP-1) ↓ ROS	Yes	
Mei 2019 [22]	Human chondrocytes (OA)		↓ ROS ↓ NOX-4 ↓ proinflammatory cytokines (IL-6 and MCP-1)	Yes	
Tong 2019 [25]	Human knee chondrocytes (OA)		↓ proinflammatory cytokines (TNF-α, IL-1β) ↓ ROS	Yes	
Chen 2018 [20]	Rats knee chondrocyte (OA)		↓ activation of NF-κB associated inflammatory cytokines (p-IκBα, p65, TNF-⍺, IL-6)		
**Animal studies**					
Que 2019 [24]	Rats	Subcutaneous liraglutide	↓ proinflammatory cytokines (TNF-⍺, IL-6, IL-1β) in cartilage tissue		Weight loss
***Analgesic effect***					
**Animal studies**					
Meurot 2022 [23]	Mice	Intra-articular liraglutide	↓ pain (↑ PWT)	Yes	

Abbreviation: ADAMTS: A disintegrin and metalloproteinase with thrombospondin motifs, CHOP: CCAAT-enhancer-binding-protein homologous protein, COX-2: cyclooxygenase-2, ECM: extracellular matrix, ER: endoplasmic reticulum, GAG: glycosaminoglycans, GRP78: glucose-regulated protein 78 ​kDa, HMG-1: high mobility group-1 protein, IκBα: Nuclear factor of kappa light polypeptide gene enhancer in B-cells inhibitor alpha, IL: interleukin, iNOS: inducible nitric oxide synthase, MMP: matrix metalloproteinases, NF-κB: nuclear factor kappa-light-chain-enhancer of activated B cells, NOX-4: nicotinamide adenine dinucleotide phosphate oxidase-4, OA: osteoarthritis, OARSI: Osteoarthritis Research Society International grade, PDI: protein disulfide isomerase, PGE2: prostaglandin E2, PWT: paw withdrawal threshold, p65: transcription factor p65, RA: rheumatoid arthritis, ROS: reactive oxygen species, TNF: tumour necrosis factor.

FOOTNOTES: When a particular effect is not reported in the Table this is because the animal or cell experiment did not describe assessment of that outcome. The table does not include additional specific outcome measures evaluated in individual studies where no significant modulation by GLP-1 agonists was observed in the particular model and time point studied.

### 4.3 Effect of GLP-1 agonists on immunomodulation

A total of seven studies evaluated the effect of GLP-1 agonists on immunomodulation [[20], [21], [22], [23], [24], [25], [26]] (Table 1 and Appendix 1 eTable 1). GLP-1 agonists were shown across six cell studies and one animal study to consistently downregulate the expression of pro-inflammatory cytokines, such that levels of interleukin (IL) -1β, IL-6, IL-8, IL-12, tumour necrosis factor-α (TNFα), HMGB-1 and/or MCP-1 were significantly reduced in chondrocytes, cartilage or synovial tissues treated with GLP-1 agonists, regardless of the type of cells, with a dose-dependent effect [[21], [22], [23],^25^], as shown in Table 1. Additionally, intracellular ROS, an inflammatory mediator that promotes degradation of the articular extracellular matrix leading to the destruction of collagen tissue in articular cartilage, in conjunction with or without NOX-4, was consistently reduced by GLP-1 agonist exposure in a dose-dependent manner, as shown in cell studies [21,22,25].

### 4.4 Effect of GLP-1 agonists on pain

There was only one animal study that demonstrated the analgesic effect of a GLP-1 agonist (Liraglutide) in osteoarthritis [23]. Liraglutide was shown to reduce pain in osteoarthritic mice, where treated animals demonstrated higher paw withdrawal threshold in response to mechanical stimulation in a dose-dependent manner, irrespective of the duration of Liraglutide administration (short term, 10-day duration or long term, 14-day duration) [23].

### 4.5 Molecular pathways through which GLP-1 agonists affects osteoarthritis

A total of five studies assessed the main cellular and molecular effector pathways whereby GLP-1 agonists modulates osteoarthritis [[20], [21], [22],^24^,^25^] (Table 2 and Appendix 1 eTable 1). Overall, pre-clinical studies suggested that the beneficial effects of GLP-1 agonists in osteoarthritis were primarily mediated through inhibiting the activation of the NF-κB pathway [21,22,25]. The nuclear translocation of p65, a subunit of the NF-κB transcription factor played a critical role in the activation of NF-κB signalling and was shown to be downregulated by GLP-1 agonists [21,25]. Additionally, there was evidence from cell studies suggesting that GLP-1 agonists suppressed the activation of several cellular responses to stress and inflammation, including the p38 mitogen-activated protein kinase (MAPK) [25] pathway and protein kinase A (PKA)/cAMP Response Element-Binding Protein (CREB) pathway [24], all leading to downstream anti-inflammatory effects.

**Table 2:** Molecular pathways of GLP-1 agonist effects in osteoarthritis in pre-clinical studies.

Author	Cell phenotype/Animal	Main findings	Dose dependent effect
**Cell studies**			
Li 2020 [21]	Human SW1353 chondrocytes (OA)	↓ nuclear translocation of p65 ↓ activation of NF-κB	Yes
Mei 2019 [22]	Human chondrocytes (OA)	↓ activation of NF-κB	Yes
Tong 2019 [25]	Human knee chondrocytes (OA)	↓ p-p38 ↓ nuclear translocation of p65 ↓ activation of NF-κB	Yes
Chen 2018 [20]	Rats knee chondrocyte	↑ GLP-1R in degenerative cartilage chondrocytes ↑ PI3K/Akt signalling in chondrocytes	Yes
**Animal studies**			
Que 2019 [24]	Rats (OA)	↑ PKA/CREB pathway ↑ GLP-1R in cartilage	

Abbreviation: CREB: cyclic adenosine monophosphate response element-binding protein, GLP-1R: glucagon-like peptide-1 receptor, IκBα: Nuclear factor of kappa light polypeptide gene enhancer in B-cells inhibitor alpha, JNK: c-Jun N-terminal kinases, NF-κB: nuclear factor kappa-light-chain-enhancer of activated B cells, OA: osteoarthritis, PI3k/Akt: phosphoinositide-3-kinase, PKA: protein kinase A, p-p38 MAPK: phosphorylated p38 mitogen-activated protein kinase, p65: transcription factor p65, RA: rheumatoid arthritis.

FOOTNOTES: When a particular effect is not reported in the Table this is because the animal experiment did not describe assessment of that outcome. The table does not include additional specific outcome measures evaluated in individual studies where no significant modulation by GLP-1 agonists was observed in the particular model and time point studied.

## 5 Human studies

### 5.1 Overall description of included studies

As shown in Table 3 and Appendix 1 eTable 2, there were 4 studies evaluating the effect of GLP-1 agonists in osteoarthritis in humans: one cohort study [15]; one post-hoc analysis of a randomised controlled trial [27] and two randomised controlled trials [28,29]. The cohort study utilised data from Shanghai Osteoarthritis Cohort (SOC) study which recruited participants through 4 hospitals in Shanghai, China [15]. Both the post-hoc analysis [27] and one of the clinical randomised controlled trials [28] recruited participants from an osteoarthritis outpatient clinic at a Danish Institute, while the other clinical randomised controlled trial was a multicenter study conducted across 11 countries [The Semaglutide Treatment Effect in People with Obesity (STEP) 9 trial] [29]. Participants from the post-hoc analysis were recruited from randomised controlled trial to determine the efficacy and safety of liraglutide in patients who are overweight or obese and have knee osteoarthritis [27]. All 4 human studies had >100 participants [15,[27], [28], [29]], with the cohort study from SOC having the most participants (n ​= ​1807). These studies recruited predominantly women (65–81.6 ​%), with mean age ranging from 56 to 60.7 years [15,[27], [28], [29]].

**Table 3:** Effect of GLP-1 agonists on structural changes and symptoms in human studies.

Author/Study	Type of OA	Main findings
***Structural effect***		
Zhu 2023 [15] Cohort study	Knee	GLP-1RA users had lower incidence of knee surgery (composite of total knee arthroplasty, uni-compartmental knee arthroplasty, arthroscopic procedures and high tibial osteotomy) compared to non-users, mainly mediated by weight reduction. GLP-1RA users had significantly lower MRI evidence of cartilage-loss velocity of the medial femorotibial joint, compared to non-users.
***Symptomatic effect***		
Bliddal 2024 [29] RCT	Knee	Semaglutide significantly reduce pain related to knee osteoarthritis, and body weight reduction, and was associated with improved physical function, as compared to placebo, over 68 weeks.
Zhu 2023 [15] Cohort study	Knee	GLP-1RA users had lower total WOMAC and pain subscale scores, and required fewer number of intra-articular steroid injections, compared to non-GLP-1RA users.
Bartholdy 2022 [27] Post-hoc analysis of RCT	Knee	GLP-1RA group had improved mean KOOS function score than the placebo group, but no difference between groups for changes in physical activity over 1 year.
Gudbergsen 2021 [28] RCT	Knee	Liraglutide had no effect on knee pain (no between group difference in KOOS pain score) despite greater weight loss in treatment group as compared to placebo group.

Abbreviation: KOOS: Knee Injury and Osteoarthritis Outcome Score, MRI: Magnetic resonance imaging, RCT: Randomised controlled trial, WOMAC: Western Ontario and McMaster Universities Osteoarthritis Index.

In contrast to participants from SOC where all of them had diabetes, only 10.6 ​% of participants from Bartholdy's post hoc analysis [27] had co-morbid diabetes. While the percentage of participants with diabetes was not reported, the clinical trials excluded participants with Type 1 and Type 2 diabetes treated with glucose-lowering drugs other than metformin [28,29].

All 4 human studies evaluated the effect of GLP-1 agonists on symptomatic and radiographic knee osteoarthritis. While the diagnosis of knee osteoarthritis was based on the American College of Rheumatology criteria in 3 studies [[27], [28], [29]], the SOC cohort had orthopaedic specialists and/or sport medicine specialists diagnosis of knee osteoarthritis [15]. All 4 studies had radiographic confirmation of knee osteoarthritis, including participants with Kellgren and Lawrence (KL) grade 1 to 3 knee osteoarthritis [15,[27], [28], [29]].

In the SOC cohort, GLP-1 agonists were prescribed for diabetes management, and notably the study excluded participants who received GLP-1 agonists for less than 2 years [15]. In contrast, participants in the post hoc analysis were from the same cohort as the clinical trial where a GLP-1 agonist (Liraglutide) was used to evaluate its effect on body weight and pain in patients with knee osteoarthritis who were overweight or obese. Importantly, in this clinical trial all participants had an 8-week run in period with intensive dietary intervention to have a minimum 5 ​% body-weight weight loss [28]. The multicenter randomised clinical trial evaluated Semaglutide in persons with obesity and knee osteoarthritis [29].

In the SOC cohort, the primary outcome measure was incidence of knee surgery after enrolment in SOC over a 6-year period, defined as all surgical procedures performed to treat knee osteoarthritis, including total knee arthroplasty, uni-compartmental knee arthroplasty, arthroscopic procedures or high tibial osteotomy [15]. The secondary outcomes of this SOC cohort study included analgesic medication use, number of intra-articular therapies, Western Ontario and McMaster Universities Osteoarthritis Index (WOMAC) and magnetic resonance imaging (MRI) evidence of medial femorotibial joint cartilage thickness changes. The main outcome for Bartholdy's post hoc analysis was change in physical activity (min/day) after 1-year assessed by accelerometer, with secondary outcomes looking at physical function evaluated by a validated questionnaire, the Knee Injury and Osteoarthritis Outcome Score (KOOS), at baseline and end of study [27]. In contrast, the clinical trial by Gudbergsen evaluated changes in body weight and the KOOS pain subscale from week 0–52 as co-primary outcomes [28]. The main outcome of the multicenter clinical trial (STEP 9 study) was percentage change in body weight and change in WOMAC pain score from baseline to week 68, with physical function score as the secondary outcome. No human studies evaluated the immunomodulatory effect of GLP-1 agonists in osteoarthritis.

### 5.2 Effect of GLP-1 agonists on structural outcomes

A single cohort study from the SOC evaluated the effect of GLP-1 agonists on structural progression [15], as shown in Table 3 and Appendix 1 eTable 2. In addition to significantly lower MRI evidence of cartilage-loss velocity of the medial femorotibial joint, GLP-1 agonist users demonstrated a substantially lower incidence of knee surgery (1.7 ​% vs 5.9 ​%, p ​= ​0.014), mainly mediated by weight reduction (mediation proportion: 32.1 ​%) [15], compared to non-users.

### 5.3 Effect of GLP-1 agonists on symptoms

All 4 human studies evaluated the symptomatic effect of GLP-1 agonists on arthritis [15,[27], [28], [29]], predominantly on its analgesic effect [15,28,29]. The SOC study showed GLP-1 agonist users had lower total WOMAC and pain subscale scores (between group difference of 3.37 on a 0–100 WOMAC scale, p ​= ​0.007), such that GLP-1 agonist users required fewer number of intra-articular steroid injections, compared to non-GLP-1 agonist users. However, compared to non GLP-1 agonist users, the GLP-1 agonist users only showed a numerically, but not statistically significant trend towards lower analgesic medication use in the SOC study [15]. In contrast, the randomised controlled clinical trial showed a GLP-1 agonist (Liraglutide) had no effect on knee pain, evaluated by KOOS pain score, despite 3.9 ​kg (kg) greater weight loss in the treatment group as compared to the placebo group [28]. In the post-hoc analysis, despite a better outcome on body weight, where participants in the Liraglutide group lost 4.1 ​kg more than the placebo group, and an improved mean KOOS function score, Liraglutide did not induce changes in physical activity in those with knee osteoarthritis over 12 months [27]. Contrary to Liraglutide, the STEP 9 trial demonstrated Semaglutide, as compared to placebo, resulted in significant pain improvement by 14.1% points (p ​< ​0.001) among people with moderate-to-severe knee osteoarthritis [29]. Compared to placebo, those on Semaglutide also had significant body weight reduction by 10.5 ​% body weight, and associated with improved physical function over 68 weeks [29].

### 5.4 Risk of bias

Appendix 2 details the risk of bias assessment for all included pre-clinical and human studies. For animal studies, overall, there was unclear risk of bias in selection, performance and detection bias due to lack of reporting details. While all animal studies reported baseline characteristics of animals, random sequence generation process, allocation concealment, random housing and random outcome assessment were not mentioned in these studies. None of the studies indicated whether the animals were randomly assigned to housing during the experiment, nor did they report whether the caregivers and/or investigators were blinded to the type of intervention the animals received. Furthermore, it was not clear how the animals were chosen for outcome assessment. There was no information provided on whether assessors were blinded when evaluating important outcomes, such as the hind paw withdrawal threshold, except for the study by Meurot [23]. No study protocols were included or made available within the manuscript in all 3 animal studies. As such, the risk of selective outcome reporting was unclear, as pre-defined primary and/or secondary outcomes were not available in the manuscript.

For non-randomised human studies, the overall risk of bias was moderate for the SOC cohort study [15], due to moderate risk of bias from confounding, selection bias and bias in outcome measures. All the randomised trials (one of which was a post-hoc analysis) had low risk of bias across all 5 domains, resulting in an overall low risk of bias for these studies [[27], [28], [29]].

## 6 Discussion

The evidence for the effect of GLP-1 agonists in osteoarthritis was systematically reviewed, with qualitative evidence synthesis derived from a total of 11 studies (7 pre-clinical and 4 human studies). Limited data from pre-clinical (cell and animal) studies consistently demonstrated that GLP-1 agonists have a chondroprotective and immunomodulatory effect in osteoarthritis, with a dose-dependent effect, where its beneficial effects was found to be primarily mediated by inhibition of NF-κB activation. Limited human studies also supported the potential analgesic effect of GLP-1 agonists in osteoarthritis.

Despite significant heterogeneity in the pre-clinical studies, there was consistent data demonstrating the chondroprotective and immunomodulatory effects of GLP-1 agonists in osteoarthritis. Pre-clinical studies had shown that GLP-1 agonists attenuated catabolic factors in chondrocytes [23,26], reduced extracellular matrix degrading proteins [[20], [21], [22],^25^,^26^], and protected chondrocytes against apoptosis [20,26], hence increasing chondrocytes viability [20,26], while reciprocally enhanced anabolic proteins [[20], [21], [22],^25^,^26^], such that there were less cartilage erosion and destruction [20,23]. The main mechanism for these beneficial effects was shown to be predominantly mediated by inhibiting the activation of NF-κB pathway. To mimic cells in disease arthritis models, all *in vitro* cell studies had co-cultured relevant cells in either IL-1β, TNFα, AGE (advanced glycation end products), or thapsigargin, an endoplastic endoplasmic reticulum stress inducer. When these diseased cells were treated with GLP-1 agonists, there were consistent anti-inflammatory effects and the resultant chondroprotective effect demonstrated.

Nevertheless, there was limited translation of these *in vitro* chondroprotective effects to human studies. There was only 1 cohort study from SOC that showed GLP-1 agonist users had lower incident of knee surgery compared to non-users, mainly mediated by weight reduction. While the incidence of knee surgery was a composite of total knee arthroplasty, uni-compartmental knee arthroplasty, arthroscopic procedures and high tibial osteotomy [15], it was primarily driven by total knee arthroplasty (1.9 ​% in non-users vs 0.9 ​% in GLP-1 agonist users) and arthroscopic procedures (3.4 ​% in non-users vs 0.9 ​% in GLP-1 agonist users). This is the only human study that examined a diabetic population, who is known to have higher incidence of obesity, metabolic syndrome and more severe osteoarthritis disease progression [30], such that the overall effect would have been underestimated. Even with significant weight loss, the clinical trial failed to show an effect on improvement of knee pain in the treatment group [28]. Notably, participants on Liraglutide had shown significant amelioration of knee pain at randomisation, likely induced by the initial lead in intense dietary intervention, resulted in a mean weight reduction of 12.5 ​kg, hence limiting the potential for further symptomatic improvement. In contrast, participants in the STEP 9 trial had significant improvement in knee osteoarthritis pain with Semaglutide, and body weight reduction of 13.7 ​%, as compared to 3.2 ​% in the placebo group. As such, magnitude of weight loss is likely a main contributor to improvement in knee pain in all these clinical trials, as it led to both direct effect of reduction in mechanical stress in knee joints in addition to improvement in metabolically induced inflammation, contributing to the beneficial effects of GLP-1 agonists in osteoarthritis.

This systematic review has several limitations. Most pre-clinical studies demonstrating the efficacy of GLP-1 agonists in chondroprotection were conducted using animals or cells from animal arthritis models in otherwise healthy, non-obese, young male animals, but not in older or aged or female sex mice or rats, despite the well-recognised differences in molecular pathophysiology compared to younger animals [31]. Older animals had been shown to manifest more severe osteoarthritis than younger animals and had a more active response to joint injury, including up-regulation of matrix genes, chemokines and matrix degrading enzymes [31]. As such, it is unclear whether GLP-1 agonists would be as effective in females and older animals on structural disease modifying and symptomatic effects in arthritis. Additionally, most pre-clinical studies had unclear risk of bias owing to under-reporting. Most human studies evaluated the use of GLP-1 agonists in overweight or obese patients without diabetes [[27], [28], [29]]. While the weight loss effect from GLP-1 agonists has been well established [[8], [9], [10]], there were only modest effects of weight loss on knee osteoarthritis symptoms, and little effect on structural improvement [7,32,33], likely due to limited reversibility in established osteoarthritis. Hence the overall effect of GLP-1 agonists in osteoarthritis may have been underestimated, and its effects in early (pre-radiological) osteoarthritis is unknown. Substantial heterogeneity was observed in the included studies (population, type of GLP-1 agonist, dose, duration, and route of delivery). Findings from this systematic review cannot be generalised to other type of osteoarthritis. The use of GLP-1 agonist in obesity is relatively new (less than 10 years). This systematic review included only 11 studies due to limited studies available. As such, the findings of this systematic review need to be interpreted cautiously.

This systematic review of *in vitro*, animal, and human studies provides support for GLP-1 agonists to be a potential disease-modifying agent in arthritis. NF-κB is a pivotal transcription factor in the pathogenesis of arthritis [21,22,34]. Pre-clinical studies had consistently demonstrated that the beneficial effects of GLP-1 agonists in osteoarthritis was primarily mediated by inhibiting the NF-κB pathway [21,22,25]. NF-κB regulates the expression of pro-inflammatory cytokines, promotes the survival of inflammatory cells, and thus contributing to cartilage destruction [34]. Inhibition of NF-κB by GLP-1 agonists resulted in reduction in phosphorylation of lκBα. Together with suppression of other cellular stress response pathways (e.g. p38 MAPK and p-JNK pathways) and inhibition of B and T cells-cell proliferation, all led to a downstream effect of reduction of pro-inflammatory cytokines [9]. Additionally, there was a clear biological gradient for the effects of GLP-1 agonists in pre-clinical studies, with a dose-response relationship demonstrated for the effect of GLP-1 agonists in chondroprotection, the degree of inhibition of NF-κB and the subsequent reduction in inflammatory cytokines. As such, by combining pre-clinical and human data, this systematic review highlights the consistency of GLP-1 agonist effects in osteoarthritis that has biological plausibility.

## 7 Conclusion

There are consistent signals across limited pre-clinical and human studies to support the potential favourable structural protective, immunomodulatory and analgesic effects of GLP-1 agonists in osteoarthritis, irrespective of the quality and heterogeneity of the included studies. Given these effects are biologically plausible and supported by a demonstrable biological gradient, further high-quality human studies are needed to confirm the findings of this systematic review, as GLP-1 agonists could be a valuable therapeutic drug option for the treatment of osteoarthritis.

## Author contributions

JC: analysis and interpretation of the data; drafting of the article; final approval of the article.

TS: analysis and interpretation of the data; drafting of the article; final approval of the article.

ME: analysis and interpretation of the data; final approval of the article.

FMC: conception and design; analysis and interpretation of the data; critical revision of the article for important intellectual content; final approval of the article.

YZL: conception and design; analysis and interpretation of the data; drafting of the article; critical revision of the article for important intellectual content; final approval of the article.

## Funding source

FMC is the recipient of NHMRC Investigator Grant (APP1194829). The funder of the study had no role in the study design and conduct of the study; collection, management, analysis and interpretation of the data; preparation, review, or approval of the manuscript; and decision to submit the manuscript for publication.

## Declaration of competing interest

None.

## Footnotes

Appendix A Supplementary data to this article can be found online at https://doi.org/10.1016/j.ocarto.2025.100567.
