# Supplementary material for: Effect of glucagon-like peptide-1 receptor agonists in osteoarthritis: A systematic review of pre-clinical and human studies
Source: Osteoarthr Cartil Open. 2025 Jan 28;7(1):100567. doi: 10.1016/j.ocarto.2025.100567 (PMC11849622; doi:10.1016/j.ocarto.2025.100567)
Supplement: Multimedia component 1 [file mmc1.pdf]

Appendix 1:

eTable 1: Characteristics of included pre-clinical studies for the effect of GLP-1 agonists on structural changes, immunomodulation, symptoms and molecular pathways

• **Cell studies**					
**Author**	**Cell source (type of animal)<br>Gender<br>Age / weight**	**Cell phenotype**	**Therapy**	**Main results**	**Conclusion**
Zhang 2024[1]<br>China	Primary chondrocytes (Sprague-Dawley rats)<br>Male<br>3-month-old / 250-300g	Knee Chondrocytes<br><br>**Knee OA**	Liraglutide 200µg/mL, cultured with AGEs 200µg/mL for 24 hours	Structural effect<br>(i) mRNA levels (fold change against control)*:<br>MMP-1:<br>Liraglutide + AGEs vs AGEs, 6 vs 14, $p<0.05$<br><br>MMP-3:<br>Liraglutide + AGEs vs AGEs, 6 vs 13, $p<0.005$<br><br>MMP-13:<br>Liraglutide + AGEs vs AGEs, 7 vs 14.5, $p<0.05$<br><br>ADAMTS-4:<br>Liraglutide + AGEs vs AGEs, 6 vs 14.5, $p<0.05$<br><br>ADAMTS-5:<br>Liraglutide + AGEs vs AGEs, 5.5 vs 9, $p<0.05$<br><br>Collagen II:<br>Liraglutide + AGEs vs AGEs, 0.6 vs 0.4, $p<0.05$<br><br>Aggrecan:<br>Liraglutide + AGEs vs AGEs, 0.6 vs 0.45, $p<0.05$<br>*Estimated from figure 4 and 5 in manuscript<br><br>(ii) Chondrocytes viability**<br>TUNEL assay (%)	Liraglutide inhibits AGEs-induced catabolic factors (MMP-1, MMP-3, MMP-13, ADAMTS-4 and ADAMTS-5) while promoted anabolic factors (type II collagen and aggrecan) in primary chondrocytes.<br><br>Liraglutide suppressed AGE-s-induced apoptotic activity of chondrocytes

				Liraglutide + AGEs vs AGEs, 12 vs 34, p<0.05<br><br>Caspase 3:<br>Liraglutide + AGEs vs AGEs, 3 vs 7.5, p<0.05<br>**Estimated from figure 6 in manuscript	
				<u>Immunomodulation</u><br>ELISA protein level*<br>IL-1ß:<br>Liraglutide + AGEs vs AGEs, 40 vs 82, p<0.05<br><br>IL-6:<br>Liraglutide + AGEs vs AGEs, 50 vs 110, p<0.05<br><br>IL-12:<br>Liraglutide + AGEs vs AGEs, 40 vs 65, p<0.05<br><br>TNFα:<br>Liraglutide + AGEs vs AGEs, 900 vs 1600, p<0.05<br>*Estimated from figure 3 in manuscript	Liraglutide inhibits AGEs-induced inflammatory cytokines (IL-1ß, IL-6, IL-12 and TNFα) in primary chondrocytes
Meurot 2022[2]<br>France	Primary chondrocytes<br>(Wild type C57BL/6 mice)<br>Male<br>12 weeks	Knee Chondrocyte<br><br>**Knee OA**	Liraglutide (10 different doses, from 6.6 to 3.4µM), cultured with IL1ß 2ng/mL for 24 hours	<u>Structural effect</u><br>mRNA levels (fold change against control)*:<br>MMP-3:<br>Liraglutide 53.1nM vs vehicle, 10 vs 0.5, p<0.05<br>Liraglutide 1700nM vs vehicle, 5.0 vs 0.5, p<0.05<br><br>MMP-13:<br>Liraglutide 53.1nM vs vehicle, 5 vs 1, p<0.05<br>Liraglutide 1700nM vs vehicle, 2.5 vs 1, p<0.05<br><br>GAG release (µg/ml)*:<br>Liraglutide 50nM + IL1ß vs IL1ß alone, mean 20 vs 28, p<0.0227.<br>*Estimated from figure 7 in manuscript.	Liraglutide treatment resulted in anti-catabolic effects (reduced MMP-3 and MMP-13) and reversed IL-1β-induced increase in GAG release from the extracellular matrix of the murine chondrocytes in a dose-dependent effect.
				<u>Immunomodulation</u><br>mRNA expression (fold change against control)*:	Liraglutide treatment resulted in anti-inflammatory effects

				iNOS:<br>Liraglutide 53.1nM vs vehicle, mean 20, p<0.05<br>Liraglutide 1700nM vs vehicle, 10, p<0.05<br><br>COX-2:<br>Liraglutide 53.1nM vs vehicle, 10, p value NR<br>Liraglutide 1700nM vs vehicle, 5, p<0.05<br><br>TNFα:<br>Liraglutide 53.1nM vs vehicle, 1.2, p value NR<br>Liraglutide 1700nM vs vehicle, 0.5, p<0.05<br>*Estimated from figure 4 in manuscript.	(reduced IL1ß-induced expression of iNOS, COX-2 and TNFα) in murine chondrocytes, in a dose-dependent effect.
Li<br>2020[3]<br>China	Human SW1353 cell line chondrosarcoma chondrocytes<br>NR<br>NR	Chondrosarcoma chondrocytes<br><br>**Knee OA**	Dulaglutide<br>50 and 100µM for 24 hours	<u>Structural effect</u><br>Protein level (relative value against control)*:<br>(i) PGE2<br>Dulaglutide 50nM + AGEs vs AGEs, 580 vs 1100, p<0.01<br>Dulaglutide 100nM + AGEs vs AGEs, 300 vs 1100, p<0.01<br><br>(ii) COX-2<br>Dulaglutide 50nM + AGEs vs AGEs, 2.9x vs 5.5x, p<0.01<br>Dulaglutide 100nM + AGEs vs AGEs, 2.1x vs 5.5x, p<0.01<br><br>(iii) Type II collagen<br>Dulaglutide 50nM + AGEs vs AGEs, 0.75 vs 0.4, p<0.01<br>Dulaglutide 100nM + AGEs vs AGEs, 0.9 vs 0.4, p<0.01<br><br>(iv) MMP-3<br>Dulaglutide 50nM + AGEs vs AGEs, 60 vs 130, p<0.01<br>Dulaglutide 100nM + AGEs vs AGEs, 40 vs 130, p<0.01<br><br>(v) MMP-13<br>Dulaglutide 50nM + AGEs vs AGEs, 120 vs 220, p<0.01<br>Dulaglutide 100nM + AGEs vs AGEs, 60 vs 220, p<0.01<br><br>(vi) Aggrecan<br>Dulaglutide 50nM + AGEs vs AGEs, 0.75 vs 0.5, p<0.01<br>Dulaglutide 100nM + AGEs vs AGEs, 0.9 vs 0.5, p<0.01	Dulaglutide ameliorates AGEs-induced PGE2 production and COX-2 expression, in a dose-dependent effect.<br><br>Dulaglutide prevents AGEs-induced degradation of type Ⅱ collagen and suppresses AGEs-induced expression of MMP-3 and MMP-13 in human SW1353 chondrocytes.<br><br>Dulaglutide prevents AGEs-induced degradation of aggrecan in human SW1353 chondrocytes.

				*Estimated from figure 3 to 8 in manuscript.	
				Immunomodulation<br>Gene level (relative value against control)*:<br>(i) IL-6<br>Dulaglutide 50nM + AGEs vs AGEs, 2.8x vs 4.5x, p<0.01<br>Dulaglutide 100nM + AGEs vs AGEs, 1.9x vs 4.5x, p<0.01<br><br>(ii) IL-8<br>Dulaglutide 50nM + AGEs vs AGEs, 3.2x vs 6.0x, p<0.01<br>Dulaglutide 100nM + AGEs vs AGEs, 2.1x vs 6.0x, p<0.01<br><br>(iii) MCP-1<br>Dulaglutide 50nM + AGEs vs AGEs, 3.1x vs 6.0x, p<0.01<br>Dulaglutide 100nM + AGEs vs AGEs, 1.8x vs 6.0x, p<0.01<br><br>Protein level (relative value) of*:<br>(i) IL-6:<br>Dulaglutide 50nM + AGEs vs AGEs, 2.5x vs 4.2x, p<0.01<br>100nM + AGEs vs AGEs, 1.6x vs 4.2x, p<0.01<br><br>(ii) IL-8<br>Dulaglutide 50nM + AGEs vs AGEs, 3.2x vs 6.2x, p<0.01<br>Dulaglutide 100nM + AGEs vs AGEs, 1.9x vs 6.2x, p<0.01<br><br>(iii) MCP-1<br>Dulaglutide 50nM + AGEs vs AGEs, 2.6x vs 5.0x, p<0.01<br>Dulaglutide 100nM + AGEs vs AGEs, 1.9x vs 5.0x, p<0.01<br>*Estimated from figure 2 in manuscript.<br><br>(iv) Intracellular ROS**<br>Dulaglutide 50nM + AGEs vs AGEs, 2.5x vs 4.2x, p<0.01<br>Dulaglutide 100nM + AGEs vs AGEs, 1.7x vs 4.2x, p<0.01<br>**Estimated from figure 4 in manuscript.	Dulaglutide attenuates AGEs-induced expression and secretion of pro-inflammatory cytokines in human SW1353 chondrocytes.<br><br>Dulaglutide mitigates AGEs-induced oxidative stress in SW1353 cells, in a dose-dependent manner.

				<u>Molecular pathways</u><br>Western blot analysis (relative value)*<br>(i) Nuclear NF-κB p65<br>Dulaglutide 50nM + AGEs vs AGEs, 2.3 vs 2.5, p<0.01<br>Dulaglutide 100nM + AGEs vs AGEs, 1.4 vs 3.5, p<0.01<br><br>(ii) Luciferase activity of NF-κB<br>Dulaglutide 50nM + AGEs vs AGEs, 50 vs 90, p<0.01<br>Dulaglutide 100nM + AGEs vs AGEs, 25 vs 90, p<0.01<br>*Estimated from figure 9 in manuscript	Dulaglutide prevents AGEs-induced activation of NF-κB in human SW1353 chondrocytes.
Mei<br>2019[4]<br>China	Human articular cartilage chondrocytes<br>NR<br>NR	Human chondrocytes<br><br>**Knee OA**	Liraglutide 50 and 100nM for 24 hours	<u>Immunomodulation</u><br>mRNA level (relative value) of*:<br>(i) TNFα:<br>Lixisenatide 10nM + IL1ß vs IL1ß, 2.5 vs 4.5, p=0.0021<br>Lixisenatide 20nM + IL1ß vs IL1ß, 2.0 vs 4.5, p=0.0036<br><br>(ii) IL-6<br>Lixisenatide 10nM + IL1ß vs IL1ß, 2.8 vs 5.2, p=0.0012<br>Lixisenatide 20nM + IL1ß vs IL1ß, 2.2 vs 5.2, p=0.0089<br><br>(iii) IL-8<br>Lixisenatide 10nM + IL1ß vs IL1ß, 2.4 vs 4.2, p=0.0073<br>Lixisenatide 20nM + IL1ß vs IL1ß, 2.0 vs 4.2, p=0.0096<br>*Estimated from figure 4 in manuscript<br><br>Protein level (pg/ml) of*:<br>(i) TNFα:<br>Lixisenatide 10nM + IL1ß vs IL1ß, 2500 vs 4500, p=0.0026<br>Lixisenatide 20nM + IL1ß vs IL1ß, 1500 vs 4500, p=0.0003<br><br>(ii) IL-6<br>Lixisenatide 10nM + IL1ß vs IL1ß, 5000 vs 8000, p=0.0042<br>Lixisenatide 20nM + IL1ß vs IL1ß, 2000 vs 8000, p=0.0001<br><br>(iii) IL-8<br>Lixisenatide 10nM + IL1ß vs IL1ß, 4000 vs 6000, p=0.0063<br>Lixisenatide 20nM + IL1ß vs IL1ß, 2000 vs 6000, p=0.0005	Lixisenatide significantly reduced expression of TNF-α, IL-6 and IL-8 at both the mRNA and protein levels.

				*Estimated from figure 4 in manuscript	
				Molecular pathways<br>Western blot analysis (relative value) of:*<br>(i) p-JNK<br>Lixisenatide 10nM + IL1ß vs IL1ß, 2.5 vs 4, p=0.0037<br>Lixisenatide 20nM + IL1ß vs IL1ß, 1.4 vs 4, p=0.0011<br><br>(ii) c-Fos<br>Lixisenatide 10nM + IL1ß vs IL1ß, 2.6 vs 3.8, p=0.0022<br>Lixisenatide 20nM + IL1ß vs IL1ß, 1.5 vs 3.8, p=0.0035<br><br>(iii) c-Jun<br>Lixisenatide 10nM + IL1ß vs IL1ß, 2.0 vs 3.5, p=0.0034<br>Lixisenatide 20nM + IL1ß vs IL1ß, 1.5 vs 3.5, p=0.0067<br>*Estimated from figure 6 in manuscript.<br><br>(iv) Nuclear p65**<br>Lixisenatide 10nM + IL1ß vs IL1ß, 2.2 vs 3.5, p=0.0025<br>Lixisenatide 20nM + IL1ß vs IL1ß, 1.2 vs 3.5, p=0.0063<br><br>(v) NF-κB p65 luciferase activity**<br>Lixisenatide 10nM + IL1ß vs IL1ß, 15 vs 35, p=0.0005<br>Lixisenatide 20nM + IL1ß vs IL1ß, 5 vs 35, p=0.0016<br>**Estimated from figure 7 in manuscript	Treatment with lixisenatide significantly reduced the level of phosphorylated JNK in a dose-dependent manner.<br><br>Treatment with lixisenatide strongly inhibited protein expression of c-fos and c-Jun in a dose-dependent manner.<br><br>Lixisenatide inhibits IL-1β-induced nuclear translocation of p65 and resultant activation of NF-κB, in a dose-dependent manner.
				Molecular pathways<br>(i) Luciferase activity of NF-κB (relative value)*<br>Liraglutide 50nM + TNFα vs TNFα, 12 vs 22, p<0.01<br>Liraglutide 100nM + TNFα vs TNFα, 5 vs 22, p<0.01<br>* Estimated from figure 7 in manuscript	Liraglutide mitigates TNF-α-induced activation of NF-κB.
Tong 2019[5]<br>China	Human primary chondrocytes (from knee cartilage)<br>NR<br>NR<br>N=18	Knee chondrocyte<br><br>**Knee OA**	Endin-4 (exenatide) 10 and 20 nM for 24 hours	Structural effect<br>(i) MMP-3 (mRNA level, relative value)*<br>Exendin-4 10nM + AGEs vs AGEs, 3.2 vs 5.2, p<0.01<br>Exendin-4 20nM + AGEs vs AGEs, 2.4 vs 5.2, p<0.01<br><br>(ii) MMP-13 (mRNA level, relative value)*	Exenatide ameliorated AGE-induced expression of MMP-3 and MMP-13, AGE-induced degradation of type II collagen, expression of ADAMTS-4 and ADAMTS-5 and degradation of

				Exendin-4 10nM + AGEs vs AGEs, 3.0 vs 4.4, $p<0.01$<br>Exendin-4 20nM + AGEs vs AGEs, 2.0 vs 4.4, $p<0.01$<br>*Estimated from figure 3<br><br>(iii) Type II collagen**<br>Exendin-4 10nM + AGEs vs AGEs, 0.7 vs 0.5, $p<0.01$<br>Exendin-4 20nM + AGEs vs AGEs, 0.8 vs 0.5, $p<0.01$<br>**Estimated from figure 4<br><br>(iv) ADAMTS-4 (mRNA level, relative value)***<br>Exendin-4 10nM + AGEs vs AGEs, 4.0 vs **5**.5, $p<0.01$<br>Exendin-4 20nM + AGEs vs AGEs, 2.5 vs 5.5, $p<0.01$<br><br>(v) ADAMTS-5 (mRNA level, relative value)***<br>Exendin-4 10nM + AGEs vs AGEs, 3.5 vs 5.0, $p<0.01$<br>Exendin-4 20nM + AGEs vs AGEs, 2.0 vs 5.0, $p<0.01$<br>***Estimated from figure 5<br><br>(vi) Aggrecan (relative value)****<br>Exendin-4 10nM + AGEs vs AGEs, 0.7 vs 0.4, $p<0.01$<br>Exendin-4 20nM + AGEs vs AGEs, 0.9 vs 0.4, $p<0.01$<br>****Estimated from figure 6	aggrecan in human primary chondrocytes.
Chen 2018[6]<br>China	Chondrocytes (Sprague-Dawley rats)<br>Male<br>200-250g	Knee chondrocytes<br><br>**Knee OA**	All rats in liraglutide group were given Liraglutide SC daily, 50μg/kg/day before they were sacrificed.<br><br>(duration of liraglutide treatment not reported)	<u>Structural effect</u><br>Chondrocytes viability (%) on electron microscopy<br>Liraglutide vs Liraglutide + IL-β, on various liraglutide concentration*:<br>Liraglutide $1x10^{-7}$, 50 vs 60, $p<0.05$<br>Liraglutide $1x10^{-6}$, 50 vs 70, $p<0.01$<br>*Estimated from figure 2 in manuscript.<br><br>Liraglutide reversed IL-β-induced chondrocytes size shrinkage and reduction in numbers, on phase-contrast microscopy (Figure 2c in manuscript).<br><br>Western blot quantification of<br>Pro-apoptotic proteins**:<br>(i) Cleaved-caspase 3	Liraglutide treatment ameliorated chondrocytes apoptosis and cartilage degeneration in rat OA model.<br><br>Liraglutide had protective effect on IL-β treated chondrocytes by decreased apoptosis in knee chondrocytes, in a dose-dependent manner.<br><br>Liraglutide significantly decreased the level of pro-apoptotic protein

				IL-β vs liraglutide 100nM + IL-β, 4.5 vs 3, $p<0.05$<br>IL-β vs liraglutide 500nM + IL-β, 4.5 vs 2, $p<0.01$<br>(ii) Bax<br>IL-β vs liraglutide 100nM + IL-β, 3 vs 2, $p<0.05$<br>IL-β vs liraglutide 500nM + IL-β, 3 vs 1.5, $p<0.05$<br><br>Anti-apoptotic proteins**:<br>(i) Bcl-2<br>IL-β vs liraglutide 100nM + IL-β, 0.25 vs 0.5, $p<0.05$<br>IL-β vs liraglutide 500nM + IL-β, 0.25 vs 0.75, $p<0.01$<br>**Estimated from figure 2 in manuscript<br><br>Western blot quantification of markers of ER stress***:<br>(i) GRP78<br>IL-β vs liraglutide 100nM + IL-β, 2.5 vs 1.5, $p<0.05$<br>IL-β vs liraglutide 500nM + IL-β, 2.5 vs 1, $p<0.01$<br><br>(ii) PDI<br>IL-β vs liraglutide 100nM + IL-β, 3 vs 2, $p<0.05$<br>IL-β vs liraglutide 500nM + IL-β, 3 vs 1.2, $p<0.01$<br><br>(iii) Caspase 12<br>IL-β vs liraglutide 100nM + IL-β, 2 vs 1.5, $p<0.05$<br>IL-β vs liraglutide 500nM + IL-β, 2 vs 1.3, $p<0.05$<br><br>(iv) CHOP<br>IL-β vs liraglutide 100nM + IL-β, 3 vs 2, $p<0.05$<br>IL-β vs liraglutide 500nM + IL-β, 3 vs 1.5, $p<0.05$<br><br>Relative mRNA expression of:<br>(i) Collagen II/ β-actin (major ECM protein)<br>TG vs liraglutide + TG, 0.4 vs 0.6, $p<0.01$<br><br>(ii) MMP-3 / β-actin (ECM degrading proteins)<br>TG vs liraglutide + TG, 13 vs 3, $p<0.01$<br>***Estimated from figure 4 in manuscript	cleaved-caspase3 and Bax and increased the level of antiapoptotic protein Bcl-2, compared with IL-1β induced chondrocytes.<br><br>GLP-1R activation decreased the ECM catabolic activity in TG-treated chondrocytes.

				<u>Immunomodulation</u><br>Western blots and immune-quantification of NF-kB associated inflammatory proteins*:<br>(i) p-IkBα<br>TG vs TG + Liraglutide, 7 vs 2, $p<0.01$<br>TG vs TG + Liraglutide + siGLP-1R, 7 vs 5.8, $p<0.01$<br><br>(ii) p65<br>TG vs TG + Liraglutide, 3 vs 1.2, $p<0.01$<br>TG vs TG + Liraglutide + siGLP-1R, 3 vs 2.8, $p<0.05$<br><br>(iii) TNF-α<br>TG vs TG + Liraglutide, 4 vs 2, $p<0.01$<br>TG vs TG + Liraglutide + siGLP-1R, 4 vs 3, $p<0.05$<br><br>(iv) IL-6<br>TG vs TG + Liraglutide, 2.8 vs 1.2, $p<0.05$<br>TG vs TG + Liraglutide + siGLP-1R, 2.8 vs 2.2, $p<0.05$<br>*Estimated from figure 5 in manuscript	Liraglutide inhibited the activation of NF-κB and subsequent inflammatory response in chondrocytes.
				<u>Molecular pathways</u><br>GLP-1R expression<br>(i) Immunohistochemical staining<br>OA knee vs healthy knee (sham), Integral absorbance of GLP-1R, 500 vs 1000, $p<0.05$*<br><br>(ii) Western blot quantification<br>OA knee vs healthy knee(control), ratio of GLP-1R/GAPDH, 0.6 vs 1.0, $p<0.05$*<br>*Estimated from figure 1 in manuscript<br><br>Western blot quantification of the ratio of p-Akt/t-Akt in Liraglutide vs Liraglutide + IL-β, on various liraglutide concentration**:<br>IL-β vs liraglutide 100nM + IL-β, 2 vs 3.5, $p<0.05$<br>IL-β vs liraglutide 500nM + IL-β, 2 vs 4.5, $p<0.01$<br>**Estimated form figure 3 in manuscript	GLP-1R is mainly expressed in cartilage chondrocytes and decreases in degenerative cartilage.<br><br>The anti-apoptotic effects of GLP-1R was modulated by PI3K/Akt signalling in chondrocytes.

• **Animal studies**						
**Author**	**Animal model**<br>**Gender**<br>**Age / weight**<br>**Number**	**Arthritis model**	**Therapy**	**Start point**<br><br>**End point**	**Results**	**Conclusion**
Meurot 2022[2]<br>France	Wild type C57BL/6 mice<br>Male<br>12-weeks-old<br>N=169	Chemically induced with IA MIA<br><br>**Knee OA**	Short-term model (N=17): Liraglutide single IA injection (day 3), 1µg, 5µg, 10µg or 20µg<br><br>Long-term model (N=10): Liraglutide weekly IA injection, 20µg	Short term model:<br>D2<br>D11<br><br>Long term model:<br>D7<br>D29	<u>Structural effect</u><br>*Short-term model*, *Total synovial score**<br>Liraglutide 20µg vs saline: 2 vs 7, p<0.01<br>*Estimated from figure 3 in manuscript<br><br>Correlation between total synovitis score and von Frey test (to assess PWT) at D10, R^2 =0.91, p<0.0001.	Liraglutide reduced Krenn synovitis severity score in MIA-induced OA in mice.
					<u>Pain</u><br>*Short-term model, PWT**:<br>Liraglutide 5µg vs vehicle:<br>0.14g vs 0.10g (D2), p<0.001;<br>0.08g vs 0.03g (D10), p<0.001.<br>Liraglutide 20µg vs saline:<br>0.14g vs 0.10g (D2), p<0.001;<br>0.20g vs 0.03g (D10), p<0.001.<br><br>*Long-term model, PWT**<br>Liraglutide 20µg vs vehicle:<br>0.1g vs 0.2g (D8), p<0.001;<br>0.4g vs 0.1g (D21), p<0.001.<br>*Estimated from figure 2 in manuscript	Liraglutide attenuated pain in MIA-induced mice models of OA, with a dose-dependent response.
					<u>Weight change</u><br>No difference in weight between treatment and comparator groups in both the short-term and long-term models.	

Que<br>2019[7]<br>China	Wistar rats<br>Male<br>Adult / 200-250g<br>N=30	Chemically induced with IA MIA<br><br>**Knee OA**	Liraglutide SC daily for 28 days, 50μg/kg/day	D1<br><br>D28	<u>Immunomodulation</u><br>Liraglutide group vs saline group<br>Quantification of protein expression for:<br>TNF-α: 0.8 vs 1.4, p< *p*<0.0001<br>IL-6: 0.9 vs 1.3, *p*<0.0001<br>IL-β: 0.6 vs 1.5, *p*<0.0001	Liraglutide reduced pro-inflammatory cytokines in the cartilage tissue of MIA-induced knee OA in rats.
					<u>Molecular pathways</u><br>Liraglutide vs saline (western blot quantification*), expression for:<br>PKA: 1.1 vs 0.6, *p*<0.0001<br>p-PKA: 1.5 vs 0.8, *p*<0.0001<br>CREB: 1.2 vs 0.5, *p*<0.0001<br>p-CREB: 1.4 vs 0.8, *p*<0.0001<br>* Estimated from figure 5 in manuscript<br><br>Expression of GLP-1R in cartilage tissue<br>Liraglutide vs saline (Western blot quantification**), 1.2 vs 0.6, p<0.0001<br>**Estimated from figure 4b in manuscript	Liraglutide upregulated the PKA/CREB pathway, and GLP-1R in the cartilage of MIA-induced rat models of OA.
					<u>Weight effect</u><br>Liraglutide vs saline*<br>Week 1: 250g vs 260g, p=0.034<br>Week 2: 245g vs 265g, p<0.0001<br>Week 3: 260g vs 280g, p<0.0001<br>Week 4: 270g vs 290g, p<0.0001<br>*Estimated from figure 4 in manuscript	Liraglutide was associated with lower body weight in MIA-induced rat models of OA.
Chen<br>2018[6]<br>China	Sprague-Dawley rats<br>Male<br>NR / 200-250g<br>N=15	Surgical induced (ACL transection with medial menisci resection)<br><br>**Knee OA**	Liraglutide SC daily, 50μg/kg/day	NR<br><br>NR	<u>Structural effect</u><br>Histopathological change of matrix layer and articular structure (figure 7a in manuscript):<br>OA group: articular cartilage showed significant destruction, cartilage erosion, proteoglycan and cellular loss<br>Liraglutide group: Significant reduction in severity of cartilage degeneration on histological analysis	Liraglutide ameliorated cartilage destruction and reduced pro-apoptotic molecules in rat models of OA.

					OARSI scores quantification (at 6 weeks)*:<br>OA vs Liraglutide + OA, 10 vs 5, p<0.05<br><br>Immunohistochemical staining of cytoplasmic of*:<br>(i) CHOP (ER-stress protein)<br>OA vs Liraglutide + OA, 3800 vs 1500, p<0.01<br><br>(ii) activated-caspase 3 (pro-apoptotic protein)<br>OA vs Liraglutide + OA, 3500 vs 1200, p<0.05<br>*Estimated from figure 7 in manuscript.	

eTable 2: Characteristics of included human studies for the effect of GLP-1 agonists on structural changes, immunomodulation, symptoms and molecular pathways

Author / Study design	Population Number Female Age	Type of OA	Intervention and comparator<br><br>GLP-1 agonist treatment duration	Outcome measures (measurement instrument)	Results	Conclusion
Bliddal 2024[8] Denmark<br><br>RCT	Age >18 with BMI≥30<br>N=407<br>F: 81.6%<br>Age: 56±10	Knee OA (radiographic evidence)	Intervention (N=271): Semaglutide 2.4mg/day<br><br>Control (N=136): Placebo<br><br>Follow up for 68 weeks	Primary end point:<br>(i) Percentage change in body weight<br><br>(ii) Change in WOMAC pain score<br><br>Secondary end points:<br>(iii) Change in WOMAC physical function score<br><br>(iv) Change in SF-36 physical function score	Semaglutide vs placebo:<br>(i) -13.7% vs -3.2% (estimated difference, -10.5 percentage points), 95% CI -12.3 to -8.6, p<0.001<br><br>(ii)-41.7 points vs -27.5 points (estimated difference, -14.1 points), 95% CI -20.0 to -8.3, p<0.001<br><br>(iii) mean change -41.5 points vs -26.7 points (estimated difference, -14.9 points), 95% CI -20.4 to -9.3, p<0.001<br><br>(iv) mean change 12.0 points vs 6.5 points (estimated difference, 5.6 points), 95% CI 3.1 to 8.0, p<0.001	Semaglutide was superior to placebo in reducing pain related to knee osteoarthritis, and body weight reduction and was associated with improved physical function, over 68 weeks.
Zhu 2023[9] China<br><br>Cohort study	Age >45 with co-morbid T2DM<br>N=1807<br>F: 74.7% (GLP-1RA users) vs. 72.7% (non-users)	Knee OA (radiographic evidence, based on specialist diagnosis)	Intervention: Any GLP-1RA (N=233)<br><br>Control: Non-users (N=1574)	(i) Incidence of any knee surgery (arthroplasty, arthroscopic procedures, osteotomy)	(i) Incidence of knee surgery was lower in GLP-1RA users compared with non-users (1.7% vs. 5.9%).	GLP-1RA users had lower incidence of knee surgery, improve WOMAC pain score and less mean cartilage thickness loss, when compared to non-users.

	Age: 60.7 (8.7) (GLP-1RA users) vs. 61.2 (8.6) (non-users)		Mean treatment duration of GLP-1 receptor agonists was 4.9 years (SD 1.9)	(ii) Use of analgesic medication<br><br>(iii) Number of IA therapies<br><br>(iv) Pain (WOMAC pain subscore)<br><br>(v) MRI evidence of medial femorotibial joint cartilage thickness	(ii) No group difference in analgesic use.<br><br>(iii) Fewer annual number of IA steroids in GLP-1RA users compared with non-users (0.13 vs. 0.22 per annum; adjusted mean difference –0.087 per annum, 95% CI –0.14, –0.036).<br><br>(iv) Lower mean WOMAC pain subscore in GLP-1RA users compared with non-users (17.1 vs. 19.4; difference –3.37, 95% CI –5.79, –0.94).<br><br>(v) Lower mean cartilage loss velocity in GLP-1RA users compared with non-users (–0.05mm/year vs. –0.07mm/year; difference 0.02mm/year, 95% CI 0.002, 0.03).	
Bartholdy 2022[10] Denmark<br><br>Cohort study (Post-hoc analysis of RCT)	Age 18 – 74 with BMI ≥27 and lost >5% TBW after 8-weeks dietary intervention, with baseline accelerometer data<br>N=135	Knee OA (radiographic evidence, based on ACR criteria)	Intervention (N=66): Liraglutide 3mg/day<br><br>Control (N=69): Placebo<br><br>Follow up for 52-weeks	(i) Change in physical activity (min/day) after one year<br><br>(ii) Change in KOOS function subscale after one year (0-100 scale with 100 indicating no disability in daily life)	(i) No group difference in change in physical activity between liraglutide group compared with placebo (15.8 vs. 14.2 min/day; difference 1.6, 95% CI –16.0, 19.1).<br><br>(ii) Improved change in mean KOOS function score in liraglutide group	

	F: 65.1% (intervention) vs. 63.8% (placebo)<br>Age: 58.8 (11.3) (intervention) vs. 58.6 (9.6) (placebo)				compared with placebo (3.7 vs. –0.1; difference 3.8, 95% CI 0.9, 6.7).	
Gudbergsen 2021[11]<br>Denmark<br><br>RCT	Randomised, triple-blinded study<br>Age 18 – 74 with BMI ≥27 and lost >5% TBW after 8-weeks dietary intervention<br>N=156<br>F: 65% (intervention) vs. 64% (placebo)<br>Age: 59.2 (10.8) (intervention) vs. 59.3 (9.7) (placebo)	Knee OA (radiographic evidence, based on ACR criteria)	Intervention (N=80): Liraglutide 3mg/day<br><br>Control (N=76): Placebo<br><br>Follow up for 52-weeks	(i) Change in body weight<br><br>(ii) Change in KOOS pain subscale (0-100 scale with 100 indicating no symptoms)	(i) Greater weight loss in liraglutide group compared with placebo (–2.8kg vs. +1.2kg; difference –3.9kg, 95% CI –6.9, –1.0).<br><br>(ii) No group difference in change in mean KOOS pain subscale between liraglutide group compared with placebo (0.4 vs. –0.6; difference 0.9, 95% CI –3.9, 5.7).	

Abbreviation:
ACL: Anterior cruciate ligament
ACR: American College of Rheumatology
ADAMTS: A disintegrin and metalloproteinase with thrombospondin motifs
AGE: Advanced glycation end products
BMI: Body mass index
CHOP: CCAAT-enhancer-binding-protein homologous protein
COX-2: Cyclooxygenase-2
CREB: Cyclic adenosine monophosphate response element-binding protein
ECM: Extracellular matrix
FLS: Fibroblast-like synoviocytes
GAG: Glycosaminoglycans

GAPDH: Glyceraldehyde 3-phosphate dehydrogenase
GLP-1R: Glucagon-like peptide-1 receptor
GLP-1RA: Glucagon like peptide-1 receptor agonist
GRP78: Glucose-regulated protein 78kDa
HMG-1: High mobility group-1 protein
IA: Intra-articular
IκBα: Nuclear factor of kappa light polypeptide gene enhancer in B-cells inhibitor alpha
IL: Interleukin
iNOS: Inducible nitric oxide synthase
JNK: c-Jun N-terminal kinases
KOOS: Knee Injury and Osteoarthritis Outcome Score
LDH: Lactate dehydrogenase
MCP-1: Monocyte chemoattractant protein-1
MIA: Mono-iodo-acetate
MMP: Matrix metalloproteinases
MRI: Magnetic resonance imaging
MTT: 3-(4, 5-dimethylthiazolyl-2)-2, 5-diphenyltetrazolium bromide
NF-κB: Nuclear factor kappa-light-chain-enhancer of activated B cells
NOX-4: Nicotinamide adenine dinucleotide phosphate oxidase-4
NR: Not reported
OA: Osteoarthritis
OARSI: Osteoarthritis Research Society International grade
PDI: Protein disulfide isomerase
PGE2: Prostaglandin E2
PI3K: Phosphoinositide 3-kinase
PKA: Protein kinase A
PWT: Paw withdrawal threshold
p-p38 MAPK: Phosphorylated p38 mitogen-activated protein kinase
p65: Transcription factor p65
RA: Rheumatoid arthritis
ROS: Reactive oxygen species
SC: Subcutaneous
siGLP-1R: Small interfering RNA for GLP-1R
TBW: Total body weight
TG: Thapsigargin (endoplasmic reticulum stress inducer)
TNF: Tumor necrosis factor
T2DM: Type 2 diabetes mellitus
WOMAC: Western Ontario and McMaster Universities Osteoarthritis Index

References

1. Zhang X, Jiang J, Xu J, Chen J, Gu Y, Wu G. Liraglutide, a glucagon-like peptide-1 receptor agonist, ameliorates inflammation and apoptosis via inhibition of receptor for advanced glycation end products signaling in AGEs induced chondrocytes. BMC Musculoskeletal Disorders 2024; 25: 601.
2. Meurot C, Martin C, Sudre L, Breton J, Bougault C, Rattenbach R, et al. Liraglutide, a glucagon-like peptide 1 receptor agonist, exerts analgesic, anti-inflammatory and anti-degradative actions in osteoarthritis. Scientific Reports 2022; 12: 1567.
3. Li H, Chen J, Li B, Fang X. The protective effects of dulaglutide against advanced glycation end products (AGEs)-induced degradation of type II collagen and aggrecan in human SW1353 chondrocytes. Chemico-Biological Interactions 2020; 322: 108968.
4. Mei J, Sun J, Wu J, Zheng X. Liraglutide suppresses TNF-alpha-induced degradation of extracellular matrix in human chondrocytes: A therapeutic implication in osteoarthritis. American Journal of Translational Research 2019; 11(8): 4800-4808.
5. Tong C, Liang H, Liu X, Yuan B, Xue B, Tong Z, et al. The protective effects of exenatide against AGEs-induced articular matrix degradation in human primary chondrocytes. American Journal of Translational Research 2019; 11(4): 2081-2089.
6. Chen J, Xie JJ, Shi KS, Gu YT, Wu CC, Xuan J, et al. Glucagon-like peptide-1 receptor regulates endoplasmic reticulum stress-induced apoptosis and the associated inflammatory response in chondrocytes and the progression of osteoarthritis in rat. Cell Death Dis 2018; 9: 212.
7. Que Q, Guo X, Zhan L, Chen S, Zhang Z, Ni X, et al. The GLP-1 agonist, liraglutide, ameliorates inflammation through the activation of the PKA/CREB pathway in a rat model of knee osteoarthritis. J Inflamm (Lond) 2019; 16: 13.
8. Bliddal H, Bays H, Czernichow S, Uddén Hemmingsson J, Hjelmesæth J, Hoffmann Morville T, et al. Once-Weekly Semaglutide in Persons with Obesity and Knee Osteoarthritis. N Engl J Med 2024; 391: 1573-1583.
9. Zhu H, Zhou L, Wang Q, Cai Q, Yang F, Jin H, et al. Glucagon-like peptide-1 receptor agonists as a disease-modifying therapy for knee osteoarthritis mediated by weight loss: findings from the Shanghai Osteoarthritis Cohort. Ann Rheum Dis 2023; 82: 1218-1226.
10. Bartholdy C, Overgaard A, Gudbergsen H, Bliddal H, Kristensen LE, Henriksen M. Changes in physical activity during a one-year weight loss trial with liraglutide vs placebo in participants with knee osteoarthritis: Secondary analyses of a randomised controlled trial. Osteoarthr Cartil Open 2022; 4: 100255.
11. Gudbergsen H, Overgaard A, Henriksen M, Waehrens EE, Bliddal H, Christensen R, et al. Liraglutide after diet-induced weight loss for pain and weight control in knee osteoarthritis: a randomized controlled trial. American Journal of Clinical Nutrition 2021; 113: 314-323.
