# Supplementary material for: Effect of glucagon-like peptide-1 receptor agonists in osteoarthritis: A systematic review of pre-clinical and human studies
Source: Osteoarthr Cartil Open. 2025 Jan 28;7(1):100567. doi: 10.1016/j.ocarto.2025.100567 (PMC11849622; doi:10.1016/j.ocarto.2025.100567)
Supplement: Multimedia component 2 [file mmc2.pdf]

Appendix 2: Risk of bias assessment for

(i) Animal studies

Author/year	Selection			Performance		Detection		Attrition	Reporting	Other biases
	Sequence generation	Baseline characteristic	Allocation concealment	Random housing	Blinding	Random outcome assessment	Blinding	Incomplete outcome data	Selective outcome reporting	
Meurot 2022[1]	?	Y	?	?	Y	?	Y	?	?	Y
Que 2019[2]	?	Y	?	?	?	?	?	N	?	Y
Chen 2018[3]	Y	Y	?	?	N	?	N	?	?	?

Signalling questions: N: No, indicates high risk of bias; Y: Yes, indicates low risk of bias; ? : unclear, indicates unclear risk of bias.

(ii) Non-randomised Studies-of Interventions (ROBINS-I)

Author / year	**Domain 1:** Bias due to confounding	**Domain 2:** Bias in selection of participants into the study	**Domain 3:** Bias in classification of interventions	**Domain 4:** Bias due to deviations from intended interventions	**Domain 5:** Bias due to missing data	**Domain 6:** Bias in measurement of outcomes	**Domain 7:** Bias in selection of the reported result	**Overall risk of bias**
Zhu 2023[4]	Moderate	Moderate	Low	Low	Low	Moderate	Low	Moderate

(iii) Randomised trials, assessed using Cochrane risk-of-bias tool for randomised trials (RoB2)

Author / year	**Domain 1:** Risk of bias arising from the randomisation process	**Domain 2:** Risk of bias due to deviations from the intended intervention	**Domain 3:** Risk of bias due to missing outcome data	**Domain 4:** Risk of bias in measurement of the outcome	**Domain 5:** Risk of bias in the selection of the reported result	**Overall risk of bias**
Bliddal 2024[5]	Low	Low	Low	Low	Low	Low
Bartholdy 2022[6]	Low	Low	Low	Low	Low	Low
Gudbergsen 2021[7]	Low	Low	Low	Low	Low	Low

References

1. Meurot C, Martin C, Sudre L, Breton J, Bougault C, Rattenbach R, et al. Liraglutide, a glucagon-like peptide 1 receptor agonist, exerts analgesic, anti-inflammatory and anti-degradative actions in osteoarthritis. Scientific Reports 2022; 12: 1567.

2. Que Q, Guo X, Zhan L, Chen S, Zhang Z, Ni X, et al. The GLP-1 agonist, liraglutide, ameliorates inflammation through the activation of the PKA/CREB pathway in a rat model of knee osteoarthritis. J Inflamm (Lond) 2019; 16: 13.
3. Chen J, Xie JJ, Shi KS, Gu YT, Wu CC, Xuan J, et al. Glucagon-like peptide-1 receptor regulates endoplasmic reticulum stress-induced apoptosis and the associated inflammatory response in chondrocytes and the progression of osteoarthritis in rat. Cell Death Dis 2018; 9: 212.
4. Zhu H, Zhou L, Wang Q, Cai Q, Yang F, Jin H, et al. Glucagon-like peptide-1 receptor agonists as a disease-modifying therapy for knee osteoarthritis mediated by weight loss: findings from the Shanghai Osteoarthritis Cohort. Ann Rheum Dis 2023; 82: 1218-1226.
5. Bliddal H, Bays H, Czernichow S, Uddén Hemmingsson J, Hjelmesæth J, Hoffmann Morville T, et al. Once-Weekly Semaglutide in Persons with Obesity and Knee Osteoarthritis. N Engl J Med 2024; 391: 1573-1583.
6. Bartholdy C, Overgaard A, Gudbergsen H, Bliddal H, Kristensen LE, Henriksen M. Changes in physical activity during a one-year weight loss trial with liraglutide vs placebo in participants with knee osteoarthritis: Secondary analyses of a randomised controlled trial. Osteoarthr Cartil Open 2022; 4: 100255.
7. Gudbergsen H, Overgaard A, Henriksen M, Waehrens EE, Bliddal H, Christensen R, et al. Liraglutide after diet-induced weight loss for pain and weight control in knee osteoarthritis: a randomized controlled trial. American Journal of Clinical Nutrition 2021; 113: 314-323.
